# Semantic Associations between Signs and Numerical Categories in the Prefrontal Cortex

**DOI:** 10.1371/journal.pbio.0050294

**Published:** 2007-10-30

**Authors:** Ilka Diester, Andreas Nieder

**Affiliations:** Primate NeuroCognition Laboratory, Department of Cognitive Neurology, Hertie-Institute for Clinical Brain Research, University of Tübingen, Tübingen, Germany; Service Hospitalier Frédéric Joliot, France

## Abstract

The utilization of symbols such as words and numbers as mental tools endows humans with unrivalled cognitive flexibility. In the number domain, a fundamental first step for the acquisition of numerical symbols is the semantic association of signs with cardinalities. We explored the primitives of such a semantic mapping process by recording single-cell activity in the monkey prefrontal and parietal cortices, brain structures critically involved in numerical cognition. Monkeys were trained to associate visual shapes with varying numbers of items in a matching task. After this long-term learning process, we found that the responses of many prefrontal neurons to the visual shapes reflected the associated numerical value in a behaviorally relevant way. In contrast, such association neurons were rarely found in the parietal lobe. These findings suggest a cardinal role of the prefrontal cortex in establishing semantic associations between signs and abstract categories, a cognitive precursor that may ultimately give rise to symbolic thinking in linguistic humans.

## Author Summary

We use symbols, such as numbers, as mental tools for abstract and precise representations. Humans share with animals a language-independent system for representing numerical quantity, but number symbols are learned during childhood. A first step in the acquisition of number symbols constitutes an association of signs with specific numerical values of sets. To investigate the single-neuron mechanisms of semantic association, we simulated such a mapping process in rhesus monkeys by training them to associate the visual shapes of Arabic numerals with the numerosity of multiple-dot displays. We found that many individual neurons in the prefrontal cortex, but only a few in the posterior parietal cortex, responded in a tuned fashion to the same numerical values of dot sets and associated shapes. We called these neurons association neurons since they establish an associational link between shapes and numerical categories. The distribution of these association neurons across prefrontal and parietal areas resembles activation patterns in children and suggests a precursor of our symbol system in monkeys.

## Abstract 3

Single neurons in the primate cortex associate numerical meaning with visual signs, thus providing insight into precursor mechanisms of human symbol acquisition.

## Introduction

Humans and animals share an evolutionarily old quantity representation system that allows the estimation of set size or number of events [1]. The assessment of numerical information is advantageous for the individual's fitness. This is particularly evident in social interactions (fight or flight decisions in contests) [2], foraging (exploiting the richer food source) [3], and parenting (discrimination of offspring) [4]. Quantity representations arise spontaneously without training as has been shown numerous times in monkeys [3] and human infants [5,6], supporting the idea that numerical competence is an ontogenetically and phylogenetically early faculty. Nonverbal numerical cognition, however, is limited to approximate quantity representations [1,7] and rudimentary arithmetic operations [5,6,8,9]; precise number representations and exact calculation are beyond its reach.

In contrast, humans familiar with number symbols are able to grasp exact cardinalities and to execute even the most abstract calculations. Humans learn to use number symbols as mental tools during childhood. Prior to the utilization of signs as numerical symbols [10], long-term associations between initially meaningless shapes (that become numerals) and inherently semantic numerical categories must inevitably be established [11,12]. Associations between shapes and quantities, a necessary first step towards the utilization of number symbols in linguistic humans, can even be mastered by animals [13–16].

Several studies in humans point to the prefrontal cortex (PFC) and the intraparietal sulcus (IPS) as key structures for both non-symbolic [17,18] and symbolic quantity information [18–20]. In monkeys, it has been shown that potentially homolog brain areas are involved in processing non-symbolic numerosity [21–26]. These studies support the hypothesis of a phylogenetic precursor system in monkeys on which higher, verbal-based numerical abilities in adult humans build up [27]. If the precursor hypothesis holds true, the same network that is involved in quantity estimation in nonhuman primates should also be engaged in the association of visual shapes with numerical categories. Here, we test this prediction by investigating whether single cells associate approximate numerosity representations with symbolic-like representations, and if so, what the respective contributions of the prefrontal and parietal cortices in this mapping process could be.

To that aim, we trained monkeys to assign visual shapes to numerical categories and recorded from single cells in both candidate regions. We report that many neurons in the PFC encoded the learned numerical value of a visual shape. In contrast, such association neurons were rarely found in the parietal lobe. Overall, the results suggest that the PFC is the prime source for the linking of signs to numerical categories in monkeys and may serve as a neuronal precursor for number symbol encoding.

## Results

### Behavior

**Figure 1:**
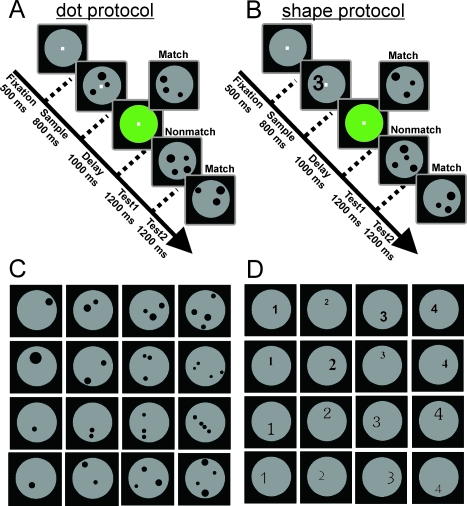
Task Protocols (A) Dot protocol designed as delayed match-to-sample task. The monkeys were required to release a lever if sample and test displays contained the same number of items, or to keep holding it otherwise (probability = 0.5). The dots' position and size were varied between sample and test display and changed in each trial. (B) Shape protocol designed as delayed association task. Task conditions were identical to those of the dot protocol, but during the sample period the numerical information was cued by an Arabic numeral. The numerals' size and position changed in each trial. (C and D) Standard (first row) and control (second to fourth row) stimuli. (C) In the dot protocol, we controlled for non-numerical cues (dot circumference, area, configuration, and density). (D) Four different fonts (“Arial” in the standard protocol; “Times New Roman,” “Souvenir BT,” and “Lithograph Light” in the controls) were presented in the shape protocol.

We trained two rhesus monkeys in a delayed match-to-sample protocol to discriminate small numerosities (one to four) in multiple-dot patterns (Figure 1A; dot protocol). The monkeys had to judge whether two successive task periods (first sample, then test) separated by a 1-s delay showed the same numerosity. If so, the animals had to release a lever. In a second step, the monkeys learned over months to associate visual shapes (Arabic numerals) with the numerosity in multiple-dot displays, i.e., Arabic numeral 1 was associated with one dot, numeral 2 was associated with two dots, and so on (Figure 1B; shape protocol). Finally, both protocols were presented in a randomly alternating fashion within a given session.

We ensured that non-numerical parameters in the dot protocol could not be used by the monkeys to solve the task by varying and controlling low-level visual features. For each session, 100 different images per numerosity were generated with pseudo-randomly varied visual properties. Sample and test stimuli were never identical. All four quantities were presented in each session with one standard and one control condition. Different control conditions were applied day by day. Controls in the dot protocol included dot displays with constant circumference, linear configuration, and constant density across all presented quantities (see Figure 1C). To force the monkeys to generalize to the overall sign characteristics in the shape protocol, the numeral shapes were varied in size, position, and font. The font “Arial” was used for the standard condition; fonts “Times New Roman,” “Souvenir BT,” and “Lithograph Light,” were used in control conditions (see Figure 1D). The test stimulus for the shape protocol consisted of sets of black dots, equivalent to the dot protocol. Trials of the standard and control conditions as well as the dot and shape protocols were pseudo-randomly intermingled and appeared with equal probabilities in each session.

**Figure 2:**
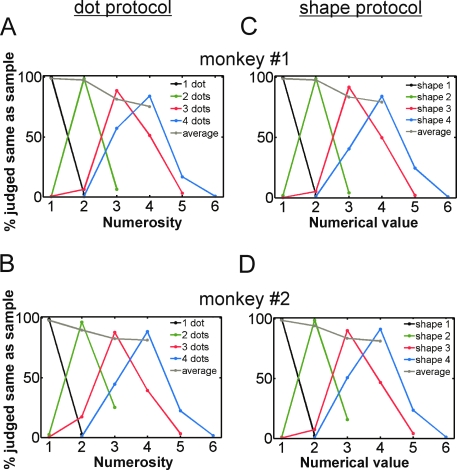
Behavioral Performance (A and B) Dot protocol. (C and D) Shape protocol. The curves show how often the monkeys judged the first test and sample numerosity to be equal. The numerical value in the first test display is shown on the *x*-axis. Each color stands for a certain numerosity shown during the sample period. Average performance for each numerosity is shown in gray as percentage correct (chance level = 50%).

Both monkeys learned reliably to associate numerical values with the visual shape of numerals. Average performance in the dot protocol (Figure 2A and 2B) and the shape protocol (Figure 2C and 2D) was comparable (87% and 88%, respectively) and significantly better than chance for all tested quantities (*p* < 0.0001, binomial test). The numerical size and distance effect [22] could be observed in both protocols, irrespective of whether the standard or control condition was applied (see Figure S1). This suggests that the monkeys were indeed judging the direct and associated numerical values.

### Neuronal Responses in PFC

**Figure 3:**
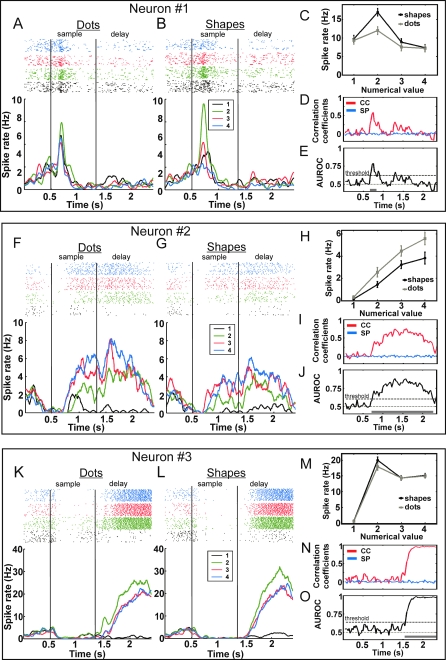
Neural PFC Responses (A–E) Neuron 1 showed highest firing rates for numerical value two in the dot (A) and shape (B) protocols in the early sample phase. Top panels in (A) and (B) show dot raster histograms (each dot represents an action potential); bottom panels are the corresponding color-coded spike density histograms (averaged and smoothed with a 100-ms Gaussian kernel for illustrative purposes only). The first 500 ms indicates the fixation period. Black vertical lines mark sample onset (500 ms) and offset (1,300 ms). (C) Tuning functions in the sample period for the dot and shape protocols, calculated from the raw firing rates in a 400-ms latency shifted window. (D) Time course of original CCs (red) and chance CCs (SPs, blue). (E) Time course of discriminability between CCs and SP quantified as the AUROC. The horizontal bar above the *x*-axis indicates the time interval of significant cross-correlation between tuning to the dot and shape protocols; in this period, the neuron associated numerical values in the two protocols. The black dashed line depicts the threshold (mean of ROC values derived during fixation period ± three standard deviations). The gray dashed line represents chance level (0.5). (F–J) Neuron 2 exhibiting four as preferred numerical value in the sample and delay period. Same layout as in (A–E); tuning functions were derived from the second ANOVA window of the sample period. (I and J) Neuron 2 associated numerical values in both protocols throughout the entire sample and delay period. (K–O) Neuron 3 exhibited two as preferred numerical value in the delay period. Same layout as in (A–E); tuning functions were derived from the second ANOVA window of the delay period.

We recorded 692 randomly selected neurons from the lateral PFC of the monkeys while they performed the tasks. Intermingled presentation of both protocols during each session allowed us to investigate individual neurons' responses to both dot and shape protocols. Many neurons were selective to numerical category and discharged strongest to specific (direct or associated) numerical values, irrespective of the protocol. Neuron 1, in Figure 3A–3E, for example, showed a maximum response to numerosity two (the neuron's preferred numerosity) in the early sample phase, and a progressive drop-off with increasing numerical distance from the preferred numerosity in the dot protocol (Figure 3A). The same neuron preferred the same (associated) numerical value (i.e., two) in the shape protocol (Figure 3B) and had an equivalent tuning function (Figure 3C). Neuron 2, in Figure 3F–3J, preferred numerosity four in both the sample and delay phase in the dot protocol (Figure 3F). The same neuron exhibited a remarkably similar temporal discharge pattern to the signs associated to specific numerical values in the shape protocol (Figure 3G). For both protocols, the neuron showed monotonically increasing tuning functions (Figure 3H). Neuron 3, in Figure 3K–3O, showed strikingly similar responses during the memory period in both the dot (Figure 3K) and shape protocol (Figure 3L), with a preferred numerical value two; the tuning functions obtained with the dot and the shape protocols were almost identical (Figure 3M).

For a quantitative analysis of the neurons' selectivity to numerical values, we first calculated a two-way analysis of variance (ANOVA) (with factors numerical value [i.e., 1, 2, 3, 4] × stimulus condition [i.e., standard versus control], *p* < 0.05) separately for the dot and shape protocols. During the sample period, 263 (263/692, or 38%) neurons were selective for shapes and 229 (229/692, or 33%) for the number of dots irrespective of whether standard or control conditions were used (significance only for factor “numerical value”; no other significant effects). During the delay period, 297 (297/692, or 43%) and 300 (300/692, or 43%) neurons were significantly tuned to shapes and the number of dots, respectively. We found 210 neurons during the sample and/or delay phase that were selective only to factor “numerical value” in both protocols, irrespective of the displays' visuospatial properties. For all quantities from one to four, we found neurons with the same preferred numerosities and associated numerical values. The observed frequency of those neurons was significantly higher compared to chance occurrence (*p* < 0.001, binomial test; Figure S5; see Materials and Methods for a description of chance level calculation). More precisely, more neurons exhibited the same preferred numerical value in the dot and shape protocols than expected assuming independence between the encoding of the two stimulus protocols.

### Neuronal Association of Visual Shapes and Numerical Values in PFC

**Figure 4:**
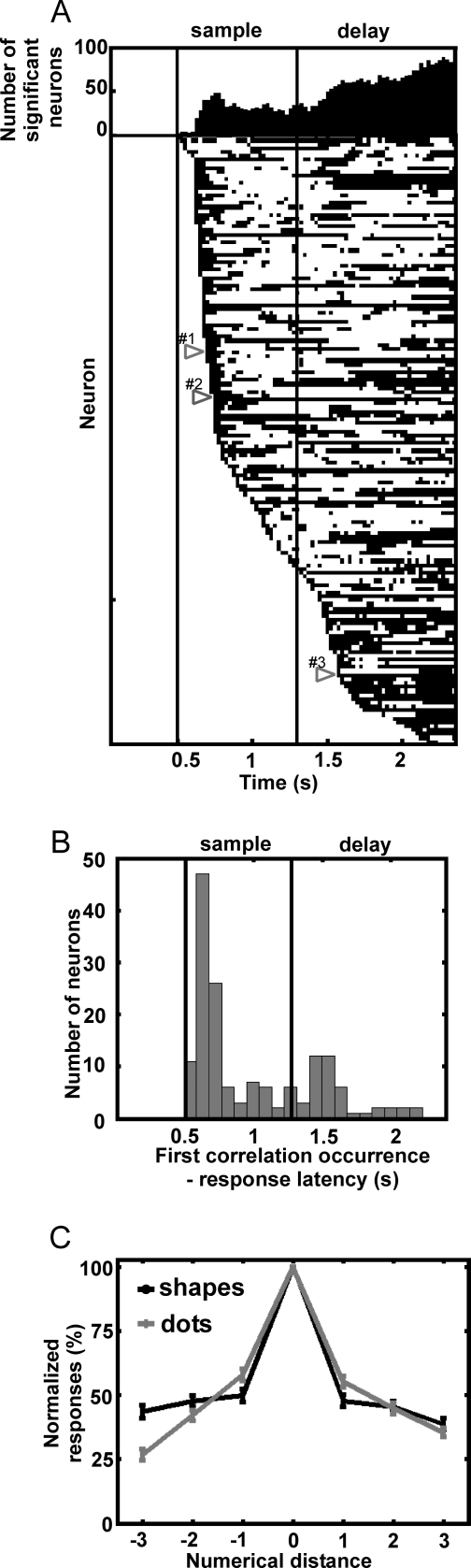
Association Neurons in the PFC (A) Diagram showing the temporal evolution of significant (as determined by a sliding ROC analysis; see Materials and Methods) cross-correlations between the tuning functions of 157 individual neurons to the dot and shape protocols. Top panel: Number of association neurons as a function of time. Bottom panel: Time course of association of each individual neuron. Each horizontal line corresponds to one single neuron. Periods of significant correlation are marked in black. Data are sorted by the first time of significant cross-correlation. Time is aligned to the midpoint of the 100-ms sliding windows. Data from example neurons 1–3 in Figure 3 are indicated by gray triangles. (B) Distribution of response-latency-corrected time points at which neurons started to associate numerical values. (C) Population tuning curves. Normalized discharges and averaged tuning curves of all association neurons to the dot (gray) and shape (black) protocols. Data are plotted as a function of numerical distance from the preferred numerical value.

Neurons that were ANOVA-selective in both protocols (especially those with the identical preferred numerical value in both protocols) constitute a potential neural substrate for long-term numerical associations. In addition to mere selectivity in the dot and shape protocols, however, neurons should have similar tuning functions for the (direct and associated) numerical values in both protocols. To test this hypothesis, and to investigate the time course of association, we performed a sliding cross-correlation analysis between each neuron's tuning functions in the shape and dot protocols for all 210 ANOVA-selective cells and derived the cross-correlation coefficients (CCs; see Figures S2–S4 for details). The significance of the CCs was evaluated by using a sliding receiver operating characteristic (ROC) analysis. For each neuron, we derived the ROC values of the difference between CCs and the shuffle predictors (SPs, which constitute chance CCs) in 25-ms time steps [28] (see Materials and Methods). Based on this analysis, 157 cells (157/692, or 23%) were significantly correlated and classified as “association neurons.” For instance, neuron 1 associated between visual shapes and numerical values during the sample onset phase, i.e., 175 ms after stimulus onset and, taking its response latency of 120 ms into account, 55 ms after its earliest visual response (Figure 3D and 3E). The associative neuronal responses of neuron 2 (Figure 3I and 3J) ranged from 250 ms (latency-corrected: 11 ms) after stimulus onset to 50 ms before the end of the delay period. As an example of a late-associating cell, neuron 3 associated throughout the entire memory phase (see Figure 3N and 3O). The time course of association shown in Figure 4A for the entire sample of association neurons revealed many neurons that associated the numerical values of shapes and dots early after sample onset. While individual cells coded the (direct and associated) numerical values during specific time phases in the trial (represented by the black bars in Figure 4A), the neuronal population represented the numerical association throughout the entire trial. When corrected for response latency, about half of the association neurons started to associate numerical values within the first 200 ms after neuronal response onset. One hundred and thirteen neurons began to associate during the sample phase, and 44 neurons during the delay phase (Figure 4B).

Interestingly, the tuning functions of association neurons showed a distance effect [22] for both protocols, i.e., a drop-off of activation with increasing numerical distance from the preferred numerical value (numerical distance 1 versus 3, dot protocol, *p* < 0.001, *n* = 104; shape protocol, *p* < 0.01, n = 91; Wilcoxon signed-rank test, two-tailed; see single-cell examples in Figure 3C, 3H, and 3M, and population analysis in Figures 4C and S6 ). The distance effect found in the shape protocol indicates that association neurons responded as a function of numerical value rather than visual shape per se. However, the neuronal response drop-off between the preferred and second-preferred numerical values was larger in the shape protocol (50%) than in the dot protocol (39.8%) (*p* = 0.016, *n* = 157, Wilcoxon signed-rank test, two-tailed). This might indicate a more precise encoding of numerical values represented by signs than by sets of dots.

### Error Trial Analysis of PFC Neurons

**Figure 5:**
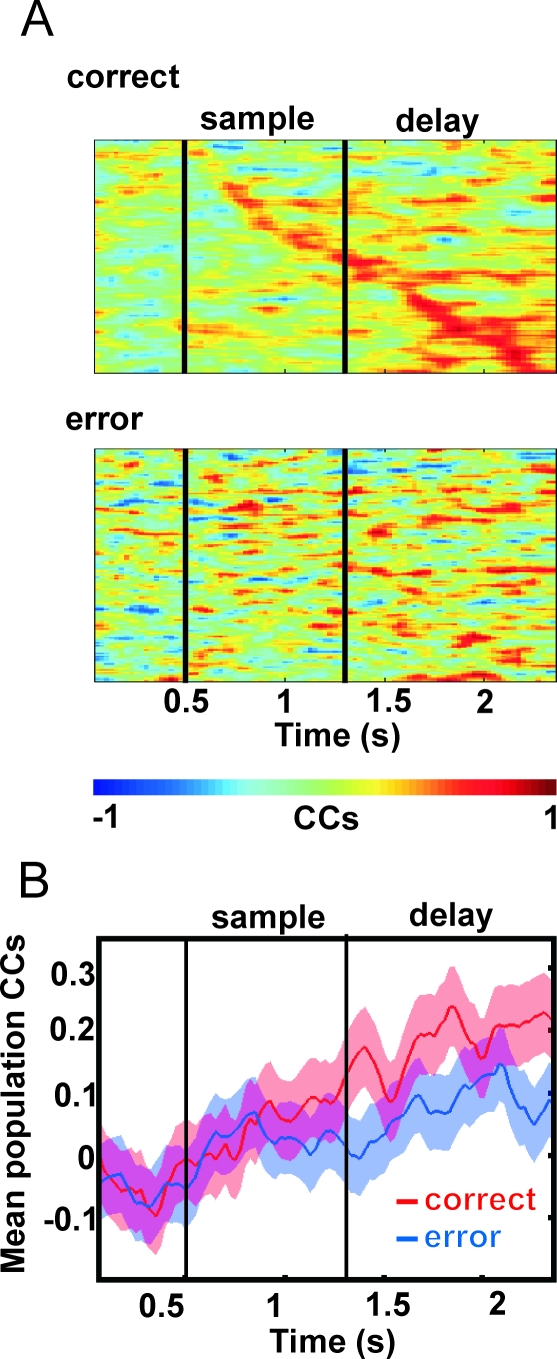
Error Trial Analysis for PFC Neurons (A) Temporal profile of CCs during correct trials (upper panel) and error trials (bottom panel) for the same association neurons (running average rectangular filter, window size five data points). Neurons are sorted by time of maximal correlation. (B) Time course of mean CCs across cells for correct and error trials (running average rectangular filter, window size five data points; shaded areas ± standard error of the mean).

Is the association of numerical values by single PFC neurons really relevant for the monkeys' behavior? If association neurons constitute a neuronal correlate for the monkeys' ability to link signs with numerosities, the tuning correlations for both protocols should be weakened whenever the monkeys failed to associate visual shapes with their corresponding numerosities in error trials. To address this issue, we calculated the CCs of association neurons between correct trials in the dot protocol and error trials in the shape protocol. Because of the monkeys' low overall error rates, error trials were only available for a subset of numerical values (e.g., 2, 3, and 4) for many neurons. Only neurons recorded during errors to two or more numerical values were included into the error trial analysis. This criterion was fulfilled by 153 out of the 157 association neurons. As shown in Figure 5A and 5B, the correlation patterns for individual neurons were disturbed in error trials, and the mean population CCs were significantly decreased in error trials during and after cue presentation (*p* < 0.001, *n* = 153, Wilcoxon signed-rank test, two-tailed). As expected, baseline correlation during the fixation period was unaffected (*p* = 0.44). These findings strongly argue for association neurons as a neuronal substrate of the semantic mapping processes between signs and categories.

### Comparison of PFC and IPS

**Figure 6:**
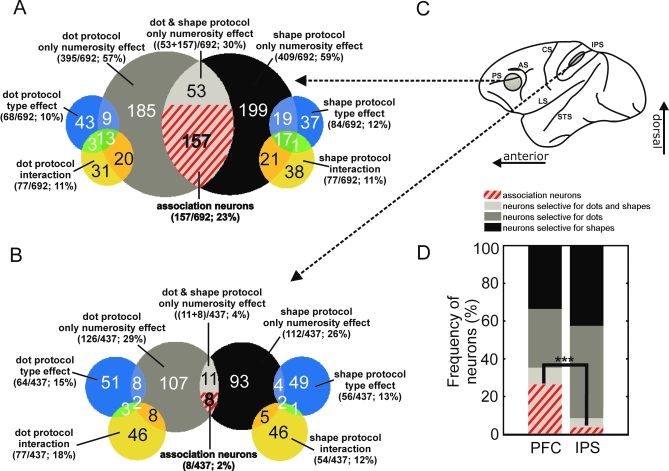
Quantitative Summary and Comparison of Neuronal Response Classes in PFC and IPS (A and B) Venn diagrams summarizing the results from the two-factor ANOVA and cross-correlation analysis in the PFC (A) and IPS (B). Data from the sample and delay period are combined. Numbers correspond to the numbers of neurons selective for each class. (C) Lateral view of a monkey brain. Circles represent schematic locations of recording sites in the frontal and parietal lobe. AS, arcuate sulcus; CS, central sulcus; PS, principal sulcus; STS, superior temporal sulcus; LS, lateral sulcus. (D) Frequency of association neurons. Proportions correspond to the added numbers of neuron classes (i.e., association neurons, ANOVA-selective neurons for both protocols, and ANOVA-selective neurons for shape or dot protocol; ***, *p* < 0.001).

During PFC recordings, we simultaneously recorded from 437 neurons in the fundus of the IPS (see Figure 6) and analyzed the neurons' responses in the same manner (i.e., two-factor ANOVA and cross-correlation analysis). In the IPS, we found many neurons encoding either the visual shapes or the numbers of dots separately (67/437, or 15%, and 62/437, or 14%, respectively, during the sample period and 58/437, or 13%, and 83/437, or 19%, respectively, during the delay period; see Figure 6B for a summary of sample and delay). The proportion of neurons showing stimulus condition and/or interaction effects in the dot and shape protocols was significantly higher in the IPS (118/437, or 27%, and 107/437, or 24%) than in the PFC (119/692, or 17%, and 133/692, or 19%) (*p* < 0.001 and *p* < 0.05, respectively; Chi-square test). This argues for a more abstract encoding of numerical values in the PFC and a more sensory-driven activity in the IPS.

In contrast to the abundance of significantly tuned IPS neurons for the shape and dot protocols, only very few IPS neurons were selectively tuned to both protocols (*n* = 19); even fewer turned out to have significant correlations (8/437; Figure 6B). Compared to the PFC, for the IPS, the proportion of association cells from the pool of all selective cells was significantly lower (*p* < 0.001, Chi-square test; Figure 6D). Nevertheless, the proportion of neurons with identical preferred numerical values in both protocols was slightly higher than expected by chance (*p* < 0.001, binomial test) (see Figure S5 and Materials and Methods for calculation of chance level and the distribution of preferred numerical values).

**Figure 7:**
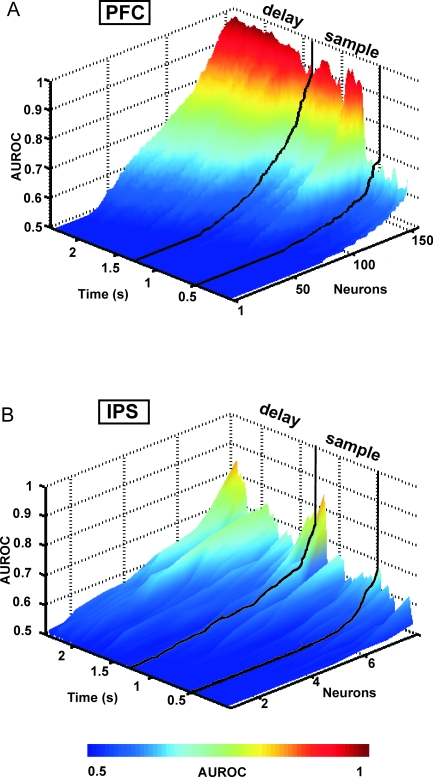
Time Course of Association Strength The association strength (measured as the AUROC) of 157 association PFC (A) and eight association IPS (B) neurons. AUROC values were sorted independently for each time bin. Higher AUROC values indicate stronger association, i.e., more similar tuning functions to the dot and shape protocols. Black lines correspond (from right to left) to sample onset and offset. Time is aligned to the midpoint of the sliding windows.

Correlation time course and correlation strength (as measured by the ROC values) were fundamentally different between PFC and IPS neurons (Figure 7). In the PFC, ROC values showed a sharp increase right after sample onset and remained elevated throughout the entire trial (Figure 7A). In the IPS, however, neuronal association was weak and occurred much later during the trial (Figure 7B); ROC values showed an increase around the end of the sample and delay period, but in contrast to values for the PFC, the IPS values were low during both periods. In summary, only PFC neurons seemed to be crucially involved in associating shapes with numerical magnitudes.

## Discussion

We trained monkeys to associate quantitative categories with inherent meaning (i.e., numerosities) with a priori meaningless visual shapes. After this long-term learning process was completed, a large proportion of PFC neurons (23%) encoded plain numerical values, irrespective of whether they had been presented as a specific number of dots or as a visual shape. The activity of association neurons predicted the monkeys' judgment performance; if the monkeys failed to match the correct number of dots to the learned shapes, discharge patterns were drastically de-correlated. The population of these PFC cells represented the numerical association throughout the entire trial, providing crucial information to bridge the association over time. In contrast, only 2% of all recorded IPS neurons associated signs with numerosities. These findings suggest the PFC as the prime source in the mapping process of visual shapes to cardinalities.

### Semantic Associations in the PFC

Previous studies showed that neurons in the PFC encode learned associations between two purely sensory stimuli without intrinsic meaning (e.g., the association of a certain color with a specific sound, or pairs of pictures) [29–31]. In the anterior inferotemporal cortex, Miyashita and co-workers found “pair-coding neurons” that responded to arbitrary pairs of images monkeys learned to match in a pair-association task [32], and evidence that the PFC is important for active retrieval of these associative representations [33]. Here we show, to our knowledge for the first time, that neurons in the PFC represent semantic long-term associations not only between pairs of pictures, but between arbitrary shapes and systematically arranged categories with inherent meaning (i.e., the ordered cardinalities of sets). Our results suggest that the PFC may not only control the retrieval of long-term associations, but may in fact constitute a cardinal processing stage for abstract semantic associations. The prefrontal region is strategically situated for such associations [34]; it receives input from both the anterior inferotemporal cortex, which encodes shape information [35], and the posterior parietal cortex, which contains numerosity-selective neurons [23,24].

The described association neurons and their response characteristics suggest such cells as neuronal correlates of semantic association. We observed that many neurons associated visual shapes with numerical values transiently, and not until the end of the delay period (Figure 4A), whereas prospective activity typically dominates near the end of the delay [29]. More importantly, a high proportion of neurons associated numerical values in the shape and dot displays right after sample onset (see Figure 4A and 4B). This argues for a direct involvement of these neurons in linking numerical values to shapes, rather than encoding upcoming match stimuli in a prospective manner. Finally, an analysis of error trials (see Figure 5) revealed that tuning correlation between both protocols was weakened whenever the monkeys failed to associate visual shapes with their corresponding numerosities. This again provides evidence that association neurons constitute a neuronal correlate for the monkeys' ability to link signs with numerosities.

### Hypothetical Formation of Association Neurons

While quantity representations are spontaneously developed [3,6], associations between visual shapes and numerical categories clearly have to be learned by mapping shape representations onto numerical categories. This neuronal learning could start with two classes of PFC cells: one class encoding visual characteristics of shapes (input possibly via inferotemporal cortex [35]), the other class representing numerical information most likely received from the IPS [23,24]. According to the Hebbian learning rule [36], the connections may be strengthened between these two classes of neurons so that cells encoding matching pairs (e.g., Arabic numeral 3 and three dots) are interconnected and become associative. This learning behavior could potentially be modeled via a recurrent neuronal network as has been done for pair-association encoding in inferotemporal neurons [37] or for somatosensory parametric working memory in PFC [38].

### Comparison of Human and Monkey Data

Even though numerosity-selective neurons in IPS are relatively abundant and encode numerical information earlier than PFC neurons [23], association neurons were surprisingly rare in the parietal lobe. Moreover, IPS neurons differentiated to a larger extent between the sensory features of the visual displays; they responded less abstractly than PFC neurons, which generalized across visual properties. At first glance, the sparseness of association IPS neurons in the nonhuman primate seems to be at odds with the well-known role of the posterior parietal cortex in adult humans for both non-symbolic [17,18] and symbolic numerical cognition [18–20]. Beyond possible species-specific differences between humans and monkeys, this difference might also be the consequence of training duration; our monkeys were trained for few months to match numerosities with visual shapes, whereas humans acquire symbols over years. Because of the monkeys' inferior proficiency, it is likely that the shape–numerosity association was not automatically executed in the monkey brain, but required a strong involvement of the PFC in order to manage the high cognitive demands [34].

Support for this assumption comes from recent functional magnetic resonance imaging studies with human children. In contrast to adults, preschoolers lacking proficiency with number symbols show elevated PFC activity when dealing with symbolic cardinalities [39–41]. Only with age and proficiency does the activation seem to shift to parietal areas. This frontal-to-parietal shift has been interpreted as being a result of increasing automaticity in number tasks. This shift of symbolic associations to the parietal lobe could release the limited cognitive resources of PFC for new demanding tasks [34]. The PFC could, thus, be ontogenetically and phylogenetically the first cortical area establishing semantic associations, which might be relocated to the parietal cortex in human adolescents [27,42] in parallel with the maturing language capabilities [43] that endow our species with a sophisticated symbolic system [42].

### A Putative Precursor for Symbolic Number Representations

During cultural evolution, humans invented number symbols as mental tools. Number symbols endow our species with an exact understanding of cardinality and the ability to execute the most complicated calculations. Given that the first ancient number symbols have been dated back to only a couple of thousand years ago [44], it is impossible that the human brain has developed areas with distinct, culturally dependent number symbol functions [27]. It is more parsimonious to assume that existing brain structures, originally evolved for other purposes, are reused and built upon in the course of continuing evolutionary development (by a process called “exaptation” [45]), an idea captured by the “redeployment hypothesis” [46] (also termed “recycling hypothesis” [27]). According to this hypothesis, already existing simpler networks are largely preserved, extended, and combined as networks become more complex, instead of there being a de novo creation of intricate structures [47]. In the number domain, evidence suggests that existing neuronal components (located in PFC and IPS)—originally developed to serve nonverbal quantity representations—are used for the new purpose of number symbol encoding, without disrupting their participation in existing cognitive processes [18]. While monkeys use the PFC and IPS for non-symbolic quantity representations [23], only the prefrontal part of this network is engaged in semantic shape–number associations. Interestingly, this pattern of brain area use seems to be preserved in human children [39–41]. Moreover, we found that numerical values represented by signs were encoded more selectively as than analog set sizes. This sharpening of the tuning functions for signs was predicted by a recent network model [48] and might indicate the advent of a digital representation via symbol-like signs in the primate brain. We speculate that our data in the monkey provide a first glimpse of redeployment of the PFC for symbolic-like learning, thus paving the way for the neuronal quantity network to encode real number symbols in language-endowed humans.

## Materials and Methods

### Behavioral protocol.

We trained two monkeys to match either a set of dots with another set of dots (delayed match-to-sample task, or dot protocol; see Figure 1A) or a visual shape with a set of dots (delayed association task, or shape protocol; see Figure 1B). Stimuli were sets of black dots or black Arabic numerals pseudo-randomly varying in size and position and displayed on a gray background. A trial started when the monkey grasped a lever and fixated (± 1.75° of visual angle, monitored with an infrared eye tracking system) on a central target. After a monkey fixated for 500 ms, the sample appeared for 800 ms (multiple-dot display in the dot protocol; Arabic numeral in the shape protocol). The monkey then had to maintain fixation until the end of a 1,000-ms delay period, after which the test stimulus was presented (always a multiple-dot pattern). In 50% of cases the test stimulus was a match, i.e., it showed the same number of dots as cued during the sample period by a multiple-dot pattern or a shape. In the other 50% of cases the first test stimulus was a nonmatch, which showed—with equal probabilities—either a higher or lower numerosity than the sample display. After a nonmatch test stimulus, a second test stimulus appeared that was always a match. To receive a fluid reward, monkeys were required to release the lever as soon as a match appeared. Trials were pseudo-randomized and balanced across all relevant features (e.g., match versus nonmatch, dot versus shape protocol, standard versus control, etc.).

### Stimuli*.*

The stimuli for the dot protocol were randomly arranged black dots displayed on a gray background (diameter 6° of visual angle). For each session, 100 different images per numerosity were generated with pseudo-randomly varied visual features: the diameter of the dots ranged from 0.5 to 0.9° of visual angle, and their positions were restricted only by the border of the gray background circle and the fact that they were not allowed to overlap each other. Sample and test stimuli were never identical. All four quantities were presented in each session with one standard and one control condition. Controls in the dot protocol included dot displays with constant circumference (the summed circumference of the dots was constant, such that dot size decreased as dot number increased, as opposed to in the standard condition), linear configuration (i.e., all dots were linearly arranged), and constant density (i.e., constant mean distance between dots) across all presented quantities (see Figure 1C). These measures prevented the monkeys from memorizing visual patterns instead of using the numerical information to solve the task. For the shape protocol, a sample stimulus consisted of a black Arabic numeral on a gray background circle. Font size (range 26 to 42 points) and position of the shapes were varied pseudo-randomly from trial to trial. The font “Arial” was used for standard trials; “Times New Roman,” “Souvenir BT,” and “Lithograph Light” were control fonts (see Figure 1D). The test stimulus for the shape protocol consisted of sets of black dots in the style of the dot protocol. Standard and control trials as well as trials from the dot and shape protocols were pseudo-randomly intermingled and appeared with equal probabilities in each session. These measures ensured that the monkeys generalized to the overall shape characteristics instead of memorizing local features.

### Recording techniques.

Recordings were made from one left and one right hemisphere of the ventral convexity of the lateral PFC and in the fundus of the IPS of two rhesus monkeys (Macaca mulatta) in accordance with the guidelines for animal experimentation approved by the Regierungspräsidium Tübingen, Germany. These areas were chosen because in preceding studies [21–26] they were shown to contain visual numerosity-selective cells and, from human studies, are known to be activated during numerosity-related tasks [17–20,39–41]. Single-cell recordings were made with arrays of tungsten electrodes (1–2 MOhm impedance). Recording sites were localized using stereotaxic reconstructions from magnetic resonance images. Recordings in the IPS were done exclusively at depths from 9 to 13 mm below the cortical surface (Horsley-Clark coordinates, anterior/posterior, −5 mm or 0 mm) [24]. No attempts were made to preselect neurons. Off-line sorting was routinely applied to separate single units. As of the publication of this article, both monkeys are still engaged in discrimination tasks.

### Response latency*.*

To determine the neuronal response latencies, averaged spike density histograms were derived with a 1-ms resolution, smoothed by a sliding window with a kernel bin width of 10 ms for all sample stimuli. A 200-ms time window before stimulus onset was used as baseline. If five consecutive time bins after stimulus onset reached a value higher than the maximum of the baseline period, response latency was defined by the first of these time bins. A default latency of 100 ms was used if no value could be calculated.

### ANOVA.

Putative association neurons were preselected based on a two-factor ANOVA. To account for different temporal response phases, spike rates were tested in four adjacent, nonoverlapping time windows. The first window (400 ms) started at the beginning of the sample period and was shifted by the neurons' response latencies. The second window (400 ms) followed right after the first one and covered the rest of the sample period. The subsequent two windows (450 ms each) covered the first and second part of the delay period. Selectivity for numerical values was calculated based on these discharge rates separately for the dot and shape protocols using a two-way ANOVA with main factors “numerical value” (one to four) and “stimulus condition” (standard and control). Cells were considered to be numerosity-selective only if they showed a significant main effect to “numerosity” in one of the four analysis windows, but no significant “stimulus condition” or interaction effect.

### Population tuning functions and normalization.

To derive averaged numerosity-filter functions, the tuning functions of individual neurons were normalized by dividing all spike rates of the tuning functions by the maximum activity, thus setting the activity at the preferred numerical value to 100%. Pooling the resulting normalized tuning curves across the entire population of association cells resulted in averaged numerosity-filter functions (see Figures 4C, S6A, and S6B). The population tuning functions were calculated for the time windows during which association neurons were significantly tuned to numerosity as tested by the two-way ANOVA. If neurons were significantly tuned in more than one window the analysis was restricted to the window with the smallest *p*-value.

### Correlation analysis.

The correlation analysis aimed to extract tuning similarities of individual neurons to numerical values in the shape and dot protocols. Figure S2 describes the application flow of the analysis. For each protocol (Figure S2A and S2B), eight trials per numerical value were chosen in a random manner (Figure S2C). Tuning functions were built with the averaged spike rates of these trials (Figure S2D and S2E). Next, the CCs between these tuning functions were calculated. The same subset of trials was shuffled so that the relation between neural activity and numerical value was abolished (Figure S2F); with this shuffled dataset, we calculated dummy tuning curves (Figure S2G and S2H) and computed the CCs (termed SPs) between them. This procedure was repeated 1,000 times, always using a new random subset consisting of eight trials to create two distributions of CCs and SPs. We quantified the discriminability between these distributions by ROC analysis. This analysis was accomplished for each of the sliding windows separately (one exemplary window is shown by the shaded bars in Figure S2A and S2B). Each separate analysis step is described in more detail below.

### Bootstrapping.

Out of the set of all trials (Figure S2A and S2B), we randomly drew eight trials per numerosity and protocol (i.e., in total four numerosities × two protocols × eight trials = 64 trials per turn; Figure S2C). This was done 1,000 times with replacement. We took care that no trial combination occurred more than once. The CCs and the SPs were calculated for each turn of the bootstrapping algorithm. This method filters robust effects across trials and provides reliable distributions.

### Tuning functions.

The tuning functions *t*_shape_ and *t*_dot_ were composed of the spike rates of a given neuron obtained in the shape and dot protocols, respectively. Spike rates were obtained by averaging across the raw spike trains for 100 ms (see shaded windows in Figure S2A and S2B). Each tuning function consisted of four spike rates (corresponding to the neuron's responses to numerical value *n* = 1, 2, 3, and 4 during the identical time window). The spike rates were combined into one tuning function by sorting them in ascending numerical order (Figure S2D and S2E).

### Cross-correlation coefficients.

The CCs provided a measure to quantify the similarity between tuning to the shape and dot protocols. The rationale behind this was the following. A neuron that was ANOVA-selective in both protocols constituted a potential neuronal association substrate between shapes and numerical values. In addition to the mere selectivity in the dot and shape protocols, however, neurons should have similar tuning functions for the (direct and associated) numerical values in both protocols. Neurons showing different tunings to the numerical values in the two protocols cannot be regarded as association neurons and should be excluded. The normalized cross-correlation is an appropriate method for filtering for these criteria. The cross-correlation takes a neuron's entire tuning functions *t*_shape_(*n*) and *t*_dot_(*n*) for the numerical values *n* ∈ [1, 2, 3, 4] for dot and shape protocols, respectively, into account, rather than just comparing the preferred numerosities. We calculated the cross-correlation between these tuning functions for the shape and dot protocols. It is scale-invariant, since the means *t̄*_shape_ and *t̄*_dot_ are subtracted from each spike rate, and has the advantage of normalization, which allows comparison across all cells. The normalized CC was calculated as follows:

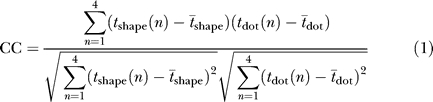


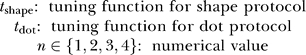


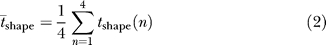


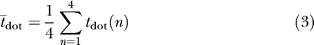



### Shuffle predictor.

The SP is supposed to represent the chance correlation level, irrespective of numerical values. For its calculation we abolished the relationship between neural activity and numerical value by randomly assigning each neural response a numerical value (Figure S2F). Based on the tuning functions of this shuffled dataset (Figure S2G and S2H), we calculated CCs. We termed the distribution of these CCs the SP. Since the SP was calculated within the bootstrapping algorithm (1,000 repetitions), it provides a robust estimate of non-numerical-related fluctuations. In other words, the SP takes accidental correlations into account (e.g., those occurring at phasic “on” responses) and can thus be regarded as baseline correlation irrespective of influences by the presented numerical values.

### ROC analysis.

To determine whether a given cell in a given time bin responded more similarly to shape and dot stimuli than expected by chance, we performed a ROC analysis [28] that provided a measure of how well the distributions of CCs and SPs were separated. The SPs were taken as the reference distribution. ROC values greater than 0.5 indicated that the CCs of a given cell were higher for the original dataset, arguing for correlated responses in the two protocols. We determined a significance threshold based on the ROC values obtained during the fixation period, during which only random correlations might occur. A neuron was termed an “association neuron” if it reached an ROC value after stimulus onset that was higher than the mean ROC value during the fixation period plus three standard deviations [49].

It needs to be emphasized that significant correlations are not caused by similar overall response modulations in the dot and shape protocols without being related to numerical value. Figure S4A and S4B shows an example neuron that responded very similarly to both protocols. Nevertheless, the CCs were close to zero (see red line in Figure S4C), because this neuron did not show any tuning to numerical value. The SP was also characterized by values fluctuating around zero (see blue line in Figure S4C). Consequently, the ROC analysis did not reveal any significant deviations from chance level (Figure S4D). In contrast, the neurons in Figure 3 showed strong modulations of firing rates with numerical value. As a consequence, the CCs reached high values up to one (see red lines in Figure 3D, 3I, and 3N). At the same time, however, the SP hovered around zero (see blue line in Figure 3D, 3I, and 3N). Thus, the ROC analysis correctly detected the periods of meaningful correlations (see Figure 3E, 3J, and 3O).

### Sliding windows.

We calculated the CCs, the SP, and the area under the ROC curve (AUROC) in sliding windows (100-ms duration, shifted by 25 ms; see shaded area in Figure S2A and S2B). This procedure allows a detailed analysis of correlation development over time (Figure 7A and 7B) and reveals the different temporal correlation patterns of individual neurons (Figure 4A). We obtained almost identical proportions of association neurons when the analysis was based on nonoverlapping windows of 100-ms duration (*n* = 167; values exceeding threshold in at least one window to reach significance).

### Error trial analysis.

We evaluated the link between neuronal responses and behavior by analyzing the influence of erroneous judgments on the neuronal association. To that aim, we calculated CCs between the neuronal tuning functions based on error trials in the shape protocol and neuronal tuning functions obtained from correct trials in the dot protocol. Since the monkeys made very few errors, we often did not collect error trials for all tested numerical values. In these cases we restricted the analysis to the numerical values for which we obtained neuronal data during error trials (at least two numerical values). We compared these error-related CCs with CCs based on correct trials (again restricted to the same numerical values).

### Probability calculation.

Was the proportion of neurons tuned to the same numerical value in both the dot and shape protocols higher than expected by chance? Since some neurons were tuned to numerosity in the dot protocol while others were encoding numerical information in the shape protocol, neurons encoding both formats may simply emerge by chance. We therefore compared the actual frequency of neurons with identical preferred numerical values in both protocols to chance occurrence based on probability calculations. To that aim, we considered the following three events: a cell is shape-selective, a cell is dot-selective, and a cell is selective for shapes and dots, formally written as

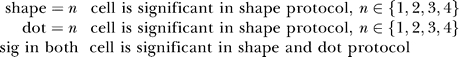
Based on our dataset, we calculated the probabilities that a cell encodes a specific preferred numerical value *n* in one of the protocols alone, given that the cell was ANOVA-selective to any numerical value in both protocols (*P*(shape *= n|*sig in both) for the shape protocol and *P*(dot *= n|*sig in both) for the dot protocol). To obtain the probability that a cell is encoding the preferred numerical value *n* in both protocols, given that the cell is selective to any numerical value in both protocols (*P*((shape = *n* ∧ dot = *n*)*|*sig in both)), the two obtained probabilities were multiplied. This approach was legitimate, because the two probabilities *P*(shape = *n|*sig in both) and *P*(dot = *n|*sig in both) are independent because of the pseudo-randomized presentation protocol. Thus, we can phrase the probability that a cell by chance encodes a specific shape and a specific number of dots simultaneously given that the cell is significant in both formats as

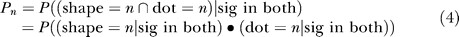
In total, the overall probability that a cell encodes one of the *n* shapes and the respective associated number of dots by chance, given that the cell is significant in both protocols, is the sum of the probabilities for all *n*:

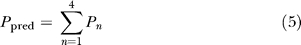
The predicted chance probability *P*_pred_ was compared to the observed probability calculated as the percentage of cells with the same preferred quantity in both protocols in the pool of cells that were ANOVA-selective in both the dot and shape protocols. We calculated binomial tests with *P*_pred_ as test proportion. The observed fractions in the PFC differed significantly from the test proportions during sample and delay period (*p* < 0.001, *n* = 93, *P*_pred_ = 0.30, and *p* < 0.001, *n* = 139, *P*_pred_ = 0.31, respectively). The fraction of neurons in the IPS with the same preferred numerical value in both protocols was very small but differed significantly from the predicted frequency during the sample and delay period (*p* < 0.001, *n* = 5, *P*_pred_ = 0.32, and *p* < 0.001, *n* = 16, *P*_pred_ = 0.25, respectively). The results are depicted as fractions of the entire sample of recorded neurons (both selective and unselective) in Figure S5E.

This analysis represents a parallel argumentation line to the cross-correlation analysis. It shows on a stochastic basis that associations of visual signs and numerical values is not a coincidence.

## Supporting Information

Figure S1 Behavioral Performance for Standard and Control Trials (A–D) Performance of monkey 1 for standard (A) and control (B) trials in the dot protocol and standard (C) and control (D) trials in the shape protocol. Same layout as in Figure 2.
(E–H) Performance of monkey 2. Layout as in (A–D).
(830 KB TIF)

Figure S2 Application Flow of Correlation Analysis (A and B) Dot raster histograms showing discharges to all trials of the dot (A) and shape (B) protocols. The numerical value is color coded.
(C) Subsets of eight randomly drawn trials per numerical value and protocol (1,000 repetitions with replacement). The numbers correspond to the numerical value shown during the sample period of the respective trial. From these trials, spike rates were calculated over 100-ms windows (indicated by the shaded time windows in [A] and [B]).
(D and E) The spike rates were arranged in a numerically ascending order for the dot (D) and shape (E) protocols, thereby forming tuning curves. With these resulting tuning curves for the dot and shape protocols, the CC was calculated.
(F–H) For the SP, we randomly shuffled discharges to the numerical values (F) and calculated the tuning functions with this shuffled dataset (G and H). With these resulting dummy tuning curves, the SP was calculated. The CCs and the SP were compared by ROC analysis.
(1.9 MB TIF)

Figure S3 Alternative Shuffling Method (A–H) Application flow of correlation analysis. Layout as in Figure S2. For the SP, we randomly shuffled average activity computed for different numerical values (F) and calculated the tuning functions with these shuffled datasets (G and H).
(I–K) Comparison of results obtained by the two different shuffling methods for the three example neurons shown in Figure 3. Upper panels show the CCs (red) and the SP (blue); lower panels illustrate the area under the ROC curve. Panels on the left represent results from the shuffling method used in this paper; panels on the right represent results from the alternative shuffling method. There are only minor differences in the results between the two shuffling methods.
(2.0 MB TIF)

Figure S4 Mere Similarities in Response Modulation Do Not Cause Significant Correlations (A and B) Dot raster and spike density histograms (100-ms smoothing Gaussian kernel) for the dot (A) and shape (B) protocols. Neuron showing similar response modulations in the dot and shape protocols, but not as a function of numerical value.
(C) The CC (red line) has values close to zero. The SP (blue line) resembles the CC.
(D) The area under the curve obtained by the ROC analysis fluctuates around 0.5. No significant correlation is detected. The black dashed lines depict the significance criterion (mean ± three standard deviations during fixation period); the gray dashed line represents the chance level.
(929 KB TIF)

Figure S5 Preferred Numerical Values of Significantly Tuned PFC and IPS Neurons (A and B) Distributions of preferred numerical values one to four in PFC neurons during sample (A) and delay (B) period. Gray and black bars correspond to the dot and shape protocols, respectively.
(C and D) Distributions of preferred numerical values in IPS neurons during sample (C) and delay (D) period.
(E) Frequency of neurons with identical preferred numerical values in both protocols. The predicted frequencies were compared with the observed data shown in (A–D) (***, *p* < 0.001). Percentages refer to the entire sample of recorded neurons (both selective and unselective).
(638 KB TIF)

Figure S6 Tuning Properties and Absolute Selectivity of PFC Association Neurons (A and B) Normalized responses averaged for neurons preferring the same sample quantity for the dot (A) and shape (B) protocols. Error bars represent the standard error of the mean.
(C) Distribution of rate differences between preferred and least preferred numerical value in the dot (gray) and shape (black) protocols for all associative neurons.
(361 KB TIF)

## Abbreviations

ANOVA: analysis of variance
AUROC: area under the receiver operating characteristic curve
CC: cross-correlation coefficient
IPS: intraparietal sulcus
PFC: prefrontal cortex
ROC: receiver operating characteristic
SP: shuffle predictor
